# Characterization of an Aptamer Directed against 25-Hydroxyvitamin D for the Development of a Competitive Aptamer-Based Assay

**DOI:** 10.3390/bios9040134

**Published:** 2019-11-13

**Authors:** Marc Prante, Torsten Schüling, Bernhard Roth, Kort Bremer, Johanna Walter

**Affiliations:** 1 Institute of Technical Chemistry, Leibniz University of Hannover, 30167 Hannover, Germany; prante@iftc.uni-hannover.de (M.P.); schueling@iftc.uni-hannover.de (T.S.); 2 Hannover Centre for Optical Technologies, Leibniz University of Hannover, 30167 Hannover, Germany; bernhard.roth@hot.uni-hannover.de (B.R.); Kort.Bremer@hot.uni-hannover.de (K.B.); 3 Cluster of Excellence PhoenixD, (Photonics, Optics, and Engineering—Innovation Across Disciplines), 30167 Hannover, Germany

**Keywords:** 25(OH)D, aptamer, biosensing, small molecules, microarray

## Abstract

Detection of the small molecule 25-hydroxyvitamin D (25(OH)D) as the most relevant marker for vitamin D supply suffers from a high variability of results using the current detection methods, such as high-performance liquid chromatography (HPLC) and immunoassays. A new detection approach using a highly specific aptamer directed against 25(OH)D was established in this study based on the target-induced dissociation (TID) sensing approach. In this work, the aptamer was investigated regarding its structural properties as well as its binding affinity by using microscale thermophoresis (MST). Moreover, complementary oligonucleotides were designed based on the aptamer structure and were evaluated in MST experiments. Binding experiments of immobilized aptamers were conducted in microarray experiments. It could be shown that the aptamer exhibited the usual B-DNA structure and did not form any G-quadruplexes. The design of complementary oligonucleotides for the TID assay identified a putative 25(OH)D binding site within the aptamer. The limit of detection of the established competitive assay was determined to be 5.4 nM, which sets the stage for the development of a biosensor system.

## 1. Introduction

Low vitamin D status in individuals leads to vitamin D deficiency and other illnesses, such as rickets. Vitamin D_3_ is obtained by exposure to sunlight, food, and dietary supplements. Hydroxylation in the liver leads to 25-hydroxyvitamin D (25(OH)D). The most appropriate indicator of vitamin D status is the detection of 25(OH)D, since it is the major circulating metabolite in the vitamin D pathway [1,2]. A variety of methods are currently used for the detection of 25(OH)D: high-performance liquid chromatography (HPLC), liquid chromatography–mass spectrometry (LC–MS), and immunoassays are the prevalent detection methods of 25(OH)D [3]. Normal 25(OH)D levels vary between 100 and 150 nM L^−1^. Concentrations lower than 75 nM L^−1^ drastically increase the risk of bone diseases such as osteoporosis and rickets [4]. Major problems in the detection process of 25(OH)D include varying test results between different detection methods [5]. Moreover, low 25(OH)D concentrations present in blood pose an additional challenge in the development of new detection methods. The detection method needs to operate between concentrations of 0 and 150 nM L^−1^ 25(OH)D in a reproducible manner [4]. Furthermore, inexpensive, fast, and easy tests are favorable. In recent years, aptamers have become molecules of choice for the detection of a wide range of targets. Aptamers are oligonucleotides that are able to form three-dimensional structures through Van der Waals interactions or electrostatic interactions and that can bind to a variety of targets [6]. Aptamers were successfully used for detecting other relevant molecules, such as β-conglutin and IgE and small molecules such as ochratoxin A, cocaine, and adenosine triphosphate (ATP) [7,8]. Besides, aptamers are considered to exhibit ideal properties for use in biomedical applications: First, aptamers are highly stable in a wide range of different environments. Second, compared to antibodies, aptamers can be produced at low cost. Last, the molecules have a long shelf life and can be regenerated after denaturation [9].

Like antibodies, aptamers can be highly specific for their designated target. Aptamers are selected via the systematic evolution of ligands by exponential enrichment (SELEX). In the selection process, the aptamer candidates can be exposed to structurally equivalent target molecules to preclude any cross-reactivity with similar molecules [10]. When used for the detection of molecules, aptamers are usually immobilized on a surface. This is due to the fact that immobilization drastically improves the handling of the sensor, and the aptamers can be regenerated more easily [11]. When incorporated in sensor platforms, the systems are referred to as aptasensors and can be considered to be biosensors. The detection principles of aptasensors can vary from optical detection principles such as surface plasmon resonance (SPR) and porous silicone (PSi)-based systems to electrochemical approaches [12,13].

**Figure 1:**
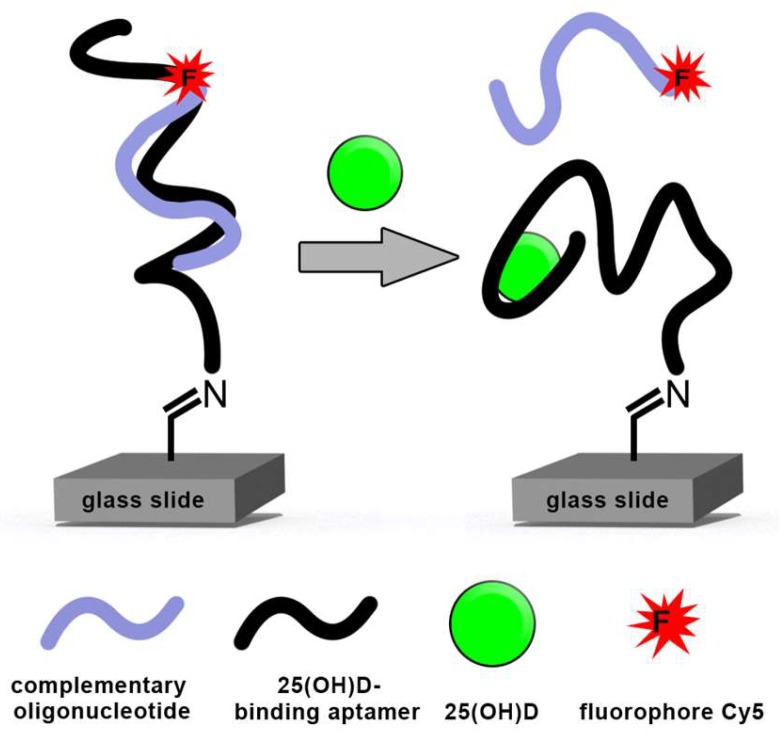
Scheme of a target-induced dissociation (TID) mode for the detection of 25-hydroxyvitamin D (25(OH)D) using fluorescence-labeled, complementary oligonucleotides and the 25(OH)D-binding aptamer VDBA14.

Moreover, due to their oligonucleotide nature, it is possible to design competitive assays through the application of complementary oligonucleotides that bind to the aptamer. The oligonucleotide can be labeled and is dissociated from the aptamer upon binding to the target molecule. Due to the small size of the target molecule (25(OH)D), a competitive assay format is intended to maximize the signal-to-noise ratio. Upon target binding, aptamers often fold into well-defined three-dimensional structures, thereby disrupting the hybridization and offering an opportunity to measure the amount of released labeled oligonucleotide (see Figure 1). This target-induced dissociation mode (TID) is often used for the detection of small molecules [14]. Aptasensors based on this mode have been successfully established for the detection of cocaine, ATP, and adenosine [8]. Another mode of operation for the detection of small molecules is real-time quantitative polymerase chain reaction (qPCR). This method is also based on the displacement of complementary oligonucleotides. The detection of ochratoxin A and ATP using this method has been successful in the nanomolar concentration range [15,16].

The single-stranded DNA-aptamer VDBA14 was selected against 25(OH)D by Lee, Nguyen, and Gu [17]. The aptamer has already been used in a gold nanoparticle aggregation-based assay, resulting in a limit of detection of 1 nM [18]. Alyamani et al. used the aptamer VDBA14 in a fluorescence-based assay and reported the ultrasensitive detection of 25(OH)D (1 fM in buffer and 200 fM in extracted blood samples) [19]. Moreover the specificity of the aptamer was proven by challenging the assays with closely related small molecules, including vitamin D_2_.

In this study, oligonucleotides complementary to different parts of the aptamer were tested for their suitability as a detection marker to design a TID mode system for the detection of 25(OH)D. In order to demonstrate the suitability of the developed system in biosensing approaches, its performance after immobilization was investigated using aptamer microarrays.

## 2. Materials and Methods

### 2.1. Chemicals and DNA Oligonucleotides

Here, 25(OH)D was purchased from Enzo Life Sciences (Farmingdale, NY, USA), dissolved in 100% ethanol, and stored at −20 °C. For the TID assay experiments, 25(OH)D was transferred in aptamer-binding buffer (100 mM NaCl, 20 mM Tris-HCl, 2 mM MgCl_2_, 5 mM KCl, 1 mM CaCl_2_, pH 7.6) with a final ethanol concentration of 10%, unless otherwise stated. In addition, 3x saline sodium citrate (SSC) was used to prepare aptamer spotting and consisted of 300 mM NaCl and 30 mM trisodium citrate. Phosphate-buffered saline (PBS; 137 mM NaCl, 2.7 mM KCl, 10 mM Na_2_HPO_4_, 1.8 mM KH_2_PO_4_) was used for the blocking solution. The single-stranded DNA aptamer VDBA14 (5′-AGC-AGC-ACA-GAG-GTC-ATG-GGG-GGT-GTG-ACT-TTG-GTG-TGC-CTA-TGC-GTG-CTA-CGG-AA-3′) was selected by Lee, Nguyen, and Gu against 25(OH)D [17]. Three complementary oligonucleotides with a length of 10 bp were designed based on the predicted aptamer structure. The oligonucleotides (O1: 5′-Cy5-TCA-CAC-CCC-C; O2: 5′-Cy5-CAC-CAA-AGT-C; and O3: 5′-Cy5-AAA-GTC-ACA-C) were purchased from Integrated DNA Technologies (Coralville, IA, USA). All oligonucleotides were dissolved in nuclease-free water and stored at −20 °C.

### 2.2. Circular dichroism (CD) Spectroscopy

The VDBA14 aptamer was diluted in aptamer-binding buffer to a final concentration of 10 μM, heated at 95 °C for 10 min, and then stored on ice for 5 min to ensure aptamer folding. The target molecule 25(OH)D was added at a concentration of 10 μM, and the aptamer–target solution was incubated for 24 h at 4 °C. CD spectroscopy measurements were carried out with a JASCO J-1000 spectrophotometer (JASCO, Tokyo, Japan) and a quartz cell (optical path length = 10 mm). The spectra were obtained through the accumulation of three scans to average the spectra from 200 to 320 nm. The scanning speed was adjusted to 50 nm min^−1^, and baselines were recorded using the respective buffer.

### 2.3. Binding Affinity Assays

Microscale thermophoresis (MST) experiments were performed with a Monolith NT.115 (NanoTemper, München, DE, Germany) and premium capillaries (NanoTemper, München, DE, Germany) to minimize adsorption: 25(OH)D was diluted in aptamer-binding buffer containing 10% ethanol starting with a concentration of 10 μM. The 5′-Cy5-labeled aptamer was then added in a constant concentration of 10 nM. The solution was incubated for 1.5 h at 25 °C to ensure that binding occurred. The solution was transferred into capillaries and measured in the Monolith NT.115. MST measurements were carried out at a temperature of 35 °C.

To verify that the used aptamer was suitable for assays based on target-induced dissociation, the designed complementary oligonucleotide was added to the aptamer–25(OH)D mixture. In this setup, the oligonucleotide was labeled 5′-Cy5, and the VDBA14 aptamer with a 5′-NH_2_ modification was used. If not stated otherwise, 10 nM oligonucleotides were used, and the rest of the experimental procedure was carried out as stated above. To investigate the effect of direct competition between the oligonucleotide and 25(OH)D, the three components were added simultaneously, incubated for 1.5 h at 25 °C, and measured afterwards. For data analysis, the program NT.Analysis Software (Nanotemper Technology GmbH, München, DE, Germany) was used. Curves were fitted using the *K_d_* model of the NT. Analysis Software and then drawn in Origin 2018 (OriginLab Corporation, Northampton, MA, USA).

### 2.4. Aptamer Immobilization

The 5′-NH_2_-modified aptamer was spotted on 3D aldehyde-modified glass microarray slides (PolyAn GmbH, Berlin, DE, Germany) utilizing a NanoSpotter (GeSiM GmbH, Großwerkmannsdorf, DE, Germany). Spotting was performed in 3x SSC by placing 6 droplets per spot. The following parameters were used for spotting: voltage 120 V and frequency 100 Hz. The spotting layout consisted of 16 blocks with 8 replicates of each aptamer concentration. Five different aptamer concentrations starting at 50 µM were serially diluted and used for spotting. After spotting, the microarrays were dried for 2 h at 25 °C followed by incubation in a high-humidity environment (70% humidity) for 24 h. The glass slide was dried at 25 °C for 3 h afterwards. The glass slide was placed in blocking solution (0.1 g NaBH_4_, 30 mL ethanol, 10 mL PBS) and incubated for 10 min at 150 rpm and 25 °C. The slides were washed three times for 10 min (50 mL) with aptamer-binding buffer to remove the blocking solution. To verify immobilization, the microarray slide was placed in a 1:10,000 SYBR I/II (Thermo Fisher Scientific, Waltham, MA, USA) solution (50 mL, 10 min) and washed three times with the aptamer-binding buffer (50 mL, 3 min), followed by ddH_2_O (50 mL, 1 min). SYBR I stains dsDNA, whereas SYBR II stains ssDNA. Since aptamers often contain single-stranded as well as double-stranded regions, this solution was better suited than only SYBR I or SYBR II. The slide was then scanned using the GenePix 4000B scanner, as stated in Section 2.6. 

### 2.5. Microarray Experiments

The VDBA14 aptamer was spotted as stated in Section 2.4. After incubation of the microarray slide in binding buffer (50 mL, 30 min), the slide was mounted in a 16-well incubation chamber (SCHOTT Nexterion, Mainz, DE, Germany), and 150 µL of an oligonucleotide–25(OH)D solution was added to every reaction chamber. The solutions were prepared previously using 50 nM of the respective complementary oligonucleotides and a serial dilution of 25(OH)D. The slide was incubated for 2.5 h with the mixture. It should be noted that this incubation time was chosen based on in-house experience with TID-based assays and could be further optimized for this particular aptamer in future work. The individual reaction chambers were washed three times with aptamer-binding buffer (5 min, 150 rpm), followed by a washing step of the whole chip in binding buffer for 5 min (150 rpm). The chip was dried and scanned as stated in Section 2.6. The shown microarray experiments were repeated three times on different days with freshly spotted aptamer microarrays. The reduction of oligonucleotide hybridization (%) was calculated using the relative fluorescence units (RFUs) at 0 µM 25(OH)D and the RFUs at the desired 25(OH)D concentration (see Equation (1)):

(1) $$\mathrm{Reduction}\;\mathrm{of}\;\mathrm{oligonucleotide}\;\mathrm{hybridization}\;\left(\%\right)=\;100\%-\;\frac{\mathrm{RFU}\left(c\right)}{\mathrm{RFU}\left(0\;µM\;25\left(\mathrm{OH}\right)D\right)}*100\%.$$

The limit of detection (LOD) was determined with the standard deviation of the blank measures (*SD_blank_*, *N* = 6) and the sensitivity of the used method (*S*). The limit of detection (LOD) was calculated with Equation (2), and the limit of quantification (LOQ) was calculated with Equation (3). Sensitivity was calculated with the slope of the Δ*RFU*/Δ*concentration curve* within the interval from 0.7 to 48 nM 25(OH)D:

(2) $$LOD=\frac{3*SD_{blank}}{S},$$

(3) $$LOQ=\frac{10*SD_{blank}}{S}.$$

### 2.6. Scanning and Analysis

Microarrays were scanned using a GenePix 4000B scanner (Molecular Devices, Sunnyvale, CA, USA). A GenePix 6.0 (Molecular Devices, Sunnyvale, CA, USA) was utilized to analyze the acquired images. The mean fluorescence at λ = 635 nm (F635) per spot of the eight replicates was calculated for every aptamer concentration and is shown in the results section.

## 3. Results

The aim of this work was the development of a competitive assay for the detection of the small molecule 25(OH)D. Therefore, the structural properties of the VDBA14 aptamer were investigated as a first step before the design of complementary oligonucleotides was carried out. As a next step, three different complementary oligonucleotides were designed and evaluated regarding their displacement from the aptamer at different 25(OH)D concentrations in solution using MST. As soon as the most suitable oligonucleotide candidate was determined, competitive assays using immobilized aptamers were carried out. We have recently used this strategy (the development of a TID assay via MST and its transfer to an aptamer–microarray format) successfully for a protein target [20]. Here, we demonstrate the suitability of the approach for aptamers directed against small molecules.

### 3.1. Structural Properties of the Used VDBA14 Aptamer

**Figure 2:**
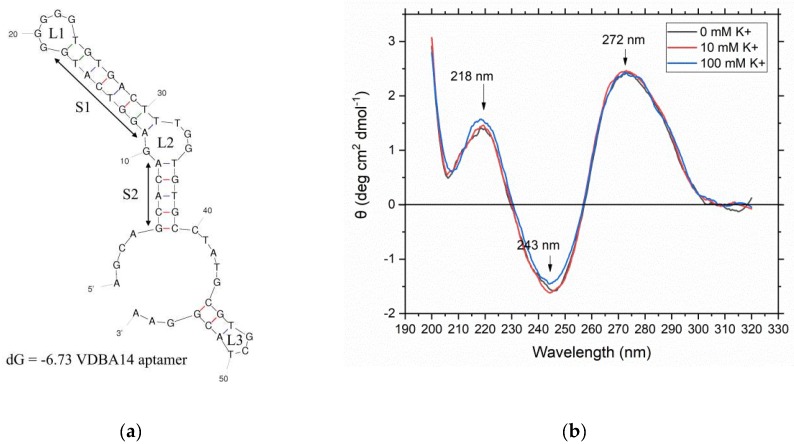
Investigation of the structural properties of the used VDBA14 aptamer. (**a**) Predicted secondary structure of the VDBA14 aptamer using mFold. Stem structures are marked as “S”, whereas loop structures are marked as “L”. The calculated initial free energy was dG = −6.73. (**b**) Circular dichroism spectrum of the VBDA14 aptamer in the presence of three different binding buffer conditions with increasing potassium concentrations (0–100 mM K^+^).

**Figure A1:**
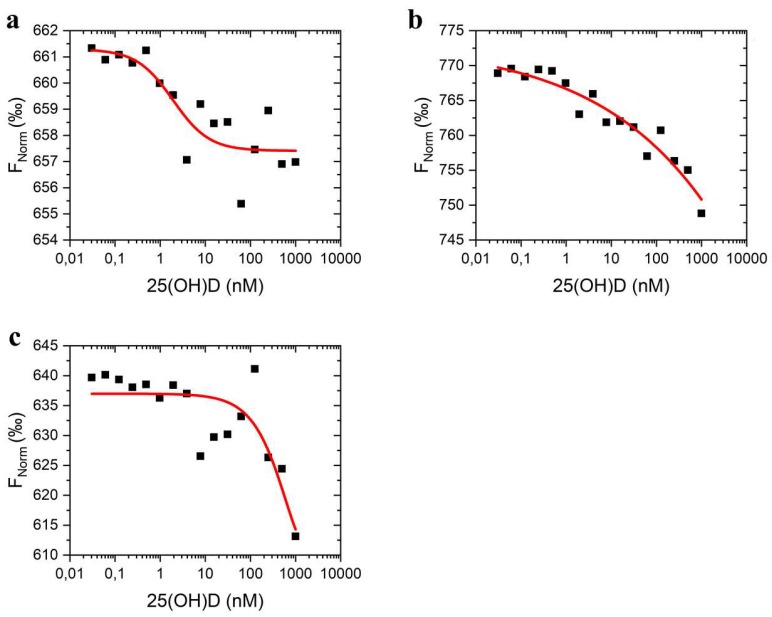
Oligonucleotides 1 (**a**), 2 (**b**), and 3 (**c**) binding to the VDBA14 aptamer at different 25(OH)D concentrations. Binding curves were generated by NT.Analysis Software and edited in Origin. The aptamer and oligonucleotide concentrations were kept constant at 50 nM, while the 25(OH)D concentration varied between 1 µM and 0.01 nM.

**Figure A2:**
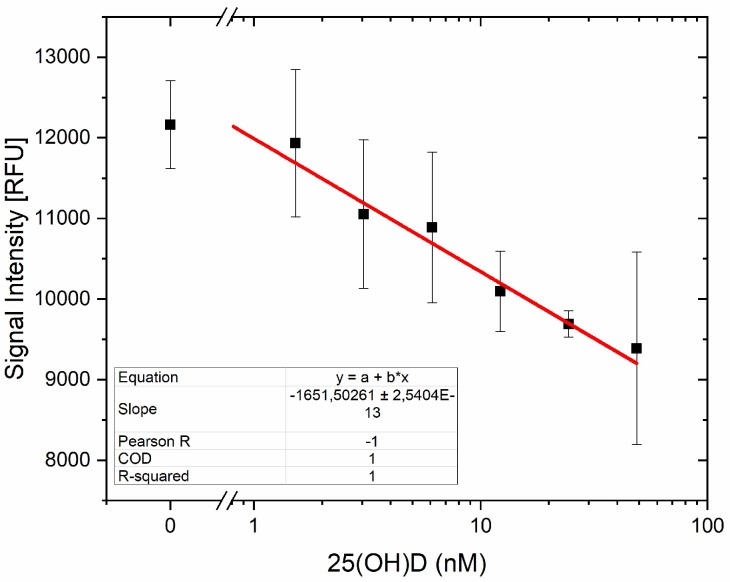
Influence of the 25(OH)D concentration on the signal intensity (relative fluorescence units (RFUs)), which was influenced by the displacement of the complementary oligonucleotide 3. The 25(OH)D concentration varied between 0 and 50 nM. The resulting data were then fitted using a linear equation in Origin. The limit of detection (LOD)/limit of quantification (LOQ) were calculated based on the sensitivity (S) of the method, here determined as the slope of the shown curve. See Section 2.5 for the equation. Error bars represent the standard deviation of three individual experiments (*N* = 3).

As a first step, the structural properties of the used VDBA14 aptamer were investigated. Therefore, the secondary structure of the aptamer was predicted using mFold, which revealed that the aptamer formed three hairpins (H1, H2, and H3) and two stems (Figure 2a, Appendix Figure A1 and Figure A2). Due to the G-rich sequence of the aptamer, the formation of intra- or intermolecular G-quadruplexes was possible. The aptamer sequence was analyzed using a QGRS mapper and scored a G-value of 14, which confirmed the possible formation of G-quadruplex structures. Aptamers that bind their targets via G-quadruplexes have been reported, but quadruplexes can be problematic in some types of assays. In particular, for assays that rely on structural changes of the aptamer upon target binding, it has to be considered that the G-quadruplex can also be formed in the absence of the target molecule and that binding of the target to a preformed quadruplex does not cause major structural rearrangements. In our envisaged assay design, which depends on the displacement of a complementary oligonucleotide from the aptamer, it also has to be considered that the high stability of quadruplexes could interfere with efficient hybridization of the oligonucleotide. Moreover, the formation of G-quadruplexes is dependent on buffer conditions, such as the presence and concentration of monovalent and divalent cations. This could result in high sensitivity of the assay toward changes in the sample composition. Due to these difficulties, which can potentially be caused by quadruplexes, we further investigated the structure of aptamer VDBA14 experimentally. 

To investigate the formation of G-quadruplexes, CD spectrometry was carried out using three different buffer conditions, as stated in Section 2.2. Parallel quadruplexes can be identified by a strong positive band at 260 nm. Antiparallel forms exhibit a dominant negative band at this wavelength and a positive one at 295 nm. Additionally, all G-quadruplexes show another positive band at 215 nm. The resulting CD spectra (Figure 2b) revealed that no G-quadruplex structures were formed, even at high potassium ion concentrations (100 mM), which strongly promote G-quadruplex formation [21].

**Figure 3:**
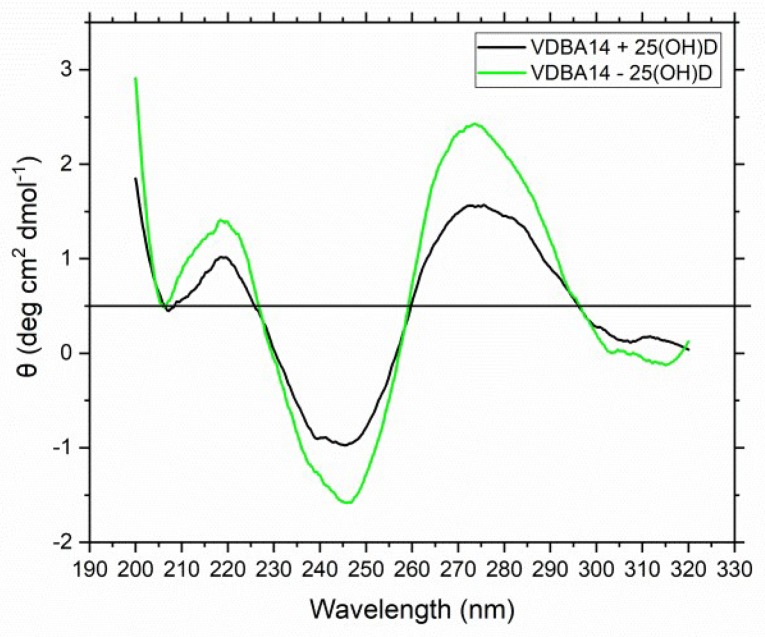
Circular dichroism spectra of the VDBA14 aptamer–25(OH)D complex (black) and the aptamer without its target (green).

Another CD spectroscopy experiment was carried out. This time, the target molecule 25(OH)D (10 μM) was added to the solution to observe structural changes of the aptamer upon target binding. Since the potassium concentration did not impact the aptamer structure, the experiment was carried out with the aptamer-binding buffer containing 5 mM potassium. The CD spectrum was recorded as stated in Section 2.2. Figure 3 shows the resulting CD spectrum for the aptamer–25(OH)D complex (black curve) and the aptamer without its target (green curve).

A comparison of the aptamer–25(OH)D complex CD spectrum to the aptamer CD spectrum revealed no shift of the wavelength spectrum upon 25(OH)D addition. This indicated that no pronounced structural reassembling of the aptamer occurred upon addition of the target molecule. However, the intensity of the CD value slightly decreased when the aptamer–25(OH)D complex formed. To further verify whether 25(OH)D is bound by the aptamer, MST experiments were carried out. 

### 3.2. Determining the K_d_ of the Used VDBA14 Aptamer

**Figure 4:**
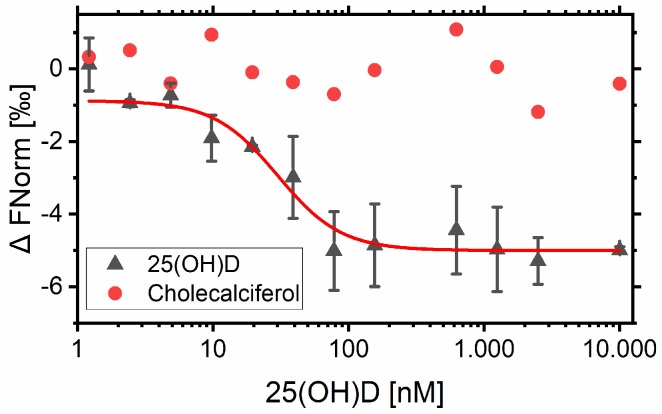
Aptamer VDBA14 binding to 25(OH)D and cholecalciferol. Binding curves were generated by NT.Analysis Software and edited in Origin. The aptamer concentration was kept constant at 10 nM, while the concentration of 25(OH)D and cholecalciferol varied from 1 nM to 10 μM. Microscale thermophoresis (MST) experiments were carried out at 35 °C. Error bars represent the standard deviation of three individual MST experiments (*N* = 3).

Cy5-labeled aptamer with a final concentration of 10 nM was added to a serial dilution of 25(OH)D ranging from 10 µM to 1 nM (see Section 2.4). The change in structure was then analyzed using MST, and the dissociation constant (*K_d_*) was determined through three replicate measurements. As a negative control, the precursor of 25(OH)D, cholecalciferol (which lacks the 25-OH group), was used in the same concentration range as 25(OH)D. The resulting binding curves are shown in Figure 4.

The *K_d_* value of the aptamer was determined to be in the nanomolar range (*K_d_* = 14.99 nM), and a relatively low response amplitude was observed (amplitude = 5). However, since 25(OH)D is a small molecule, the structural change was expected to be marginal and therefore difficult to detect in the MST. This effect could also be observed in the recorded CD spectra, where no pronounced target-induced structural change was detected. No binding could be monitored when the precursor molecule cholecalciferol was used with the VDBA14 aptamer (Figure 4, red dots). As a next step, complementary oligonucleotides were designed and evaluated with regard to their displacement at different 25(OH)D concentrations using MST. 

### 3.3. Design and Evaluation of Complementary Oligonucleotides

**Figure 5:**
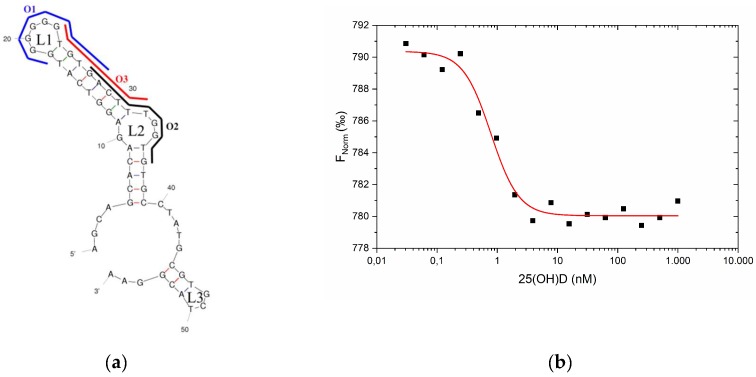
(**a**) Illustration of the predicted secondary structure of the VDBA14 aptamer, highlighting binding sites of the complementary designed oligonucleotides using mFold. (**b**) Oligonucleotide 3 binding to the VDBA14 aptamer at different 25(OH)D concentrations. For the binding curves of oligonucleotide 1 and 2, please see Figure A1. Binding curves were generated by NT.Analysis Software and edited in Origin. The aptamer and oligonucleotide concentrations were kept constant at 10 nM, while the 25(OH)D concentration varied between 1 µM and 0.01 nM.

Both the CD spectra and MST measurements indicated that no significant structural change occurred in the aptamer upon 25(OH)D binding. These results suggest that the 25(OH)D was bound in a preformed structure that could have been one of the two stems. On the basis of these findings, three complementary oligonucleotides with lengths of 10 nucleotides were designed to cover the two major loop structures (L1, L2) and the stem areas (S1 and S2) of the predicted aptamer structure as a starting point (see Figure 5a). Oligonucleotide 1 (O1) covered the G-rich loop 1, whereas oligonucleotide 2 (O2) was bound to loop 2 and was a part of S2. Oligonucleotide 3 (O3) was designed to bind the stem (S1) between the two loops. 

Here, 25(OH)D was serially diluted starting at 2 µM (1:1 dilution). The VDBA14 aptamer and the corresponding oligonucleotide were mixed previously. The aptamer–oligonucleotide solution and the 25(OH)D dilutions were then mixed and incubated for 30 min as stated in Section 2.3 with a final 25(OH)D concentration of 1 µM. The binding curves of oligonucleotides 1–2 showed low amplitudes, whereas O3 showed the largest amplitude (35,55), and target-induced dissociation could be assumed (please see Figure A1 and Table A1 for the MST data of O1–O3). However, the *K_d_* determination of O3 was vague due to a high *K_d_* confidence. On the basis of the initial results regarding amplitude values, O3 was used for the following experiments, since a stronger structural change is favored for detection in the final biosensor application. To improve the MST experiment involving O3, we optimized the O3 and aptamer concentration (now 10 nM). The apparent *K_d_* of oligonucleotide 3 with the optimized parameters (10 nM aptamer and O3, 35 °C during MST run) was determined to be 1 nM with a *K_d_* confidence of ±0.18 nM (Figure 5b).

### 3.4. Competitive Assay for the Detection of 25(OH)D

In the competitive assay format proposed for this sensor, the aptamer is immobilized on a glass slide. The labeled, complementary oligonucleotide binds to the aptamer and is displaced upon the introduction of the target molecule (see Figure 1). In the previous experiment, complementary oligonucleotides were designed and investigated in solution to determine their binding to the VDBA14 aptamer. As a next step for biosensor development, the aptamer should be immobilized on a solid surface. Therefore, the VDBA14 aptamer was immobilized on 3D aldehyde-modified microarray surfaces as stated in Section 2.4 in concentrations ranging from 50 μM to 0 μM. The immobilized aptamers were stained with a SYBR I/II solution.

**Figure 6:**
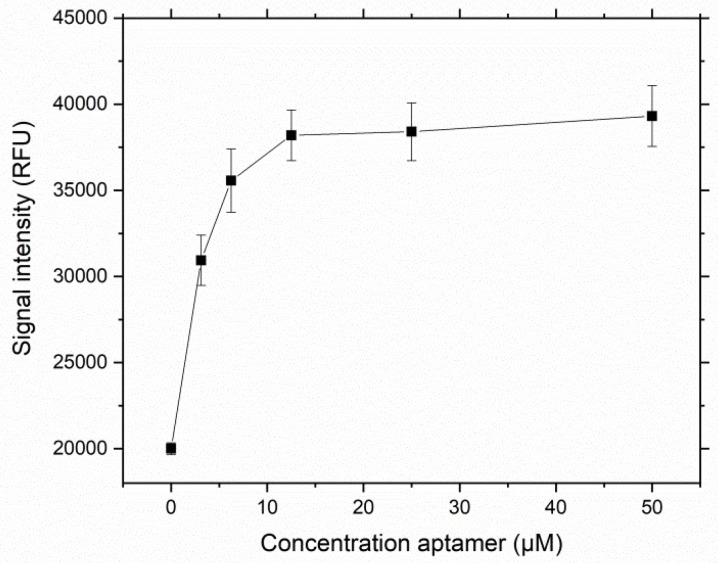
SYBR I/II staining of immobilized VDBA14 aptamer on a 3D aldehyde-modified glass slide, as stated in Section 2.4. Error bars represent the standard deviation of three individual experiments, *N* = 3.

**Figure 7:**
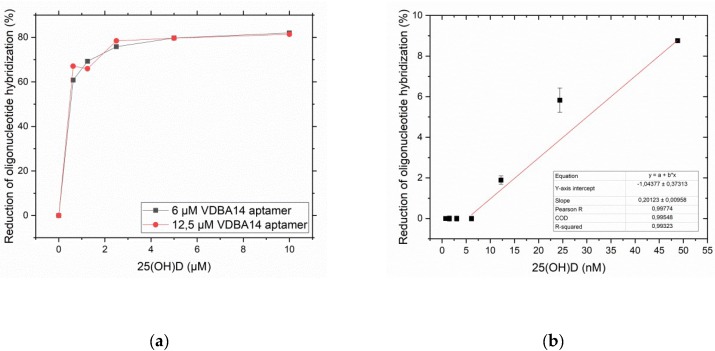
(**a**) Reduction of oligonucleotide 3 hybridization depending on the 25(OH)D concentration at two different aptamer densities (6 µM and 12.5 µM). (**b**) Reduction of oligonucleotide 3 hybridization depending on the 25(OH)D concentration range (between 0 and 50 nM). Error bars represent the standard deviation of three individual experiments (*N* = 3).

SYBR I/II staining confirmed that the immobilization of the VDBA14 on the aldehyde-modified microarray surface was successful and indicated aptamer saturation at a concentration of 12.5 μM (Figure 6). In order to investigate the aptamer functionality when immobilized, the next step was to transfer the TID assay established via MST to the microarray format. Therefore, the aptamer was immobilized on a surface at two different densities (6.25 μM, 12.5 μM). A density of 12.5 µM was chosen to achieve the highest signal intensity, and a medium density (6.25 µM) was used to avoid steric hindrance that could occur for aptamers that are immobilized in densities that are too high. The result is shown in Figure 7a. When comparing the reduction of oligonucleotide hybridization induced by 25(OH)D at the different aptamer concentrations, it is notable that the maximal reduction was reached at a 25(OH)D concentration of 2 μM. Moreover, the aptamer concentration did not seem to have an impact on the functionality of the assay, even at high immobilized aptamer densities. Additionally, this experiment confirmed the functionality of the immobilized aptamer, since the displacement of the complementary O3 could be observed with increasing 25(OH)D concentrations. Since the measuring range is important and the resolution in the 0-to-2 μM 25(OH)D range in this experiment was not sufficient for the final application, the experiment was repeated in a lower concentration range of 25(OH)D (0–50 nM).

Figure 7b shows the results of this experiment. With increasing 25(OH)D concentrations, more Cy5-labeled oligonucleotides were displaced from the VDBA14 aptamer. The LOD/LOQ were calculated as described in Section 2.5 based on the change in fluorescence intensity (RFUs) dependent on the 25(OH)D concentration. The sensitivity was determined by the slope of the linear fit (please see Figure A2 for fitting parameters). The limit of detection of the microarray system was determined to be 5.4 nM, and the limit of quantification was 18 nM.

## 4. Discussion

### 4.1. Structural Properties of the VDBA14 Aptamer

After comparing the resulting CD spectrum to different G-quadruplex spectra from the literature, it could be precluded that no G-quadruplexes but a typical B-form DNA duplex were formed by the VDBA14 aptamer. Parallel quadruplexes can be identified by a strong positive band at 260 nm. Antiparallel forms exhibit a dominant negative band at this wavelength and a positive band at 295 nm. Additionally, all G-quadruplexes show another positive band at 215 nm [22]. It has been reported in the literature that the concentration of potassium ions has a strong impact on the formation of G-quadruplexes. This effect is much stronger than the effect of sodium ions, which are also able to stabilize G-quadruplex structures through ionic interactions between the negatively charged phosphate backbones [21]. Monovalent ions such as potassium and sodium ions are located in a central channel in a quartet arrangement of four guanosines. These quartets are then stacked upon each other, and a quadruplex structure is formed. Since the K^+^ and Na^+^ concentrations of the aptamer-binding buffer were high and quadruplex forming G-rich sequences (QGRS) analysis revealed that G-quadruplexes might be formed, the aptamer structure was analyzed using CD spectroscopy. Opposite to the stabilizing nature of monovalent ions are divalent ions such as Mg^2+^ and Ca^2+^, which have a destabilizing effect on the G-quadruplex structure [21]. After investigating the structure of the aptamer, the target molecule 25(OH)D was added to the aptamer solution, and another CD spectrum was recorded. The spectrum showed no wavelength shift between the two samples, indicating that no major structural change occurred upon 25(OH)D binding. 

### 4.2. Determining the K_d_ of the VDBA14 Aptamer

After investigating the structural properties of the used VDBA14 aptamer in the previous step, the dissociation constant of the aptamer–25(OH)D complex was determined. Therefore, MST experiments were carried out, which showed a significantly different value compared to the value reported by Lee et al. via isothermal titration calorimetry (ITC) (*K_d_* = 11 nM) [17]. The *K_d_* of the used aptamer was determined to be 14.99 nM with a relatively low amplitude (amplitude = 5 AU) using MST. This was expected since the binding of the small molecule 25(OH)D leads to minimal structural changes in the aptamer. The amplitude of an MST experiment can be used as an indicator for changes in size, charge, and conformation induced by target binding [23]. Since neither the size nor the charge of the aptamer will be changed drastically upon binding with the small molecule 25(OH)D, it is reasonable to assume that the amplitude is mainly dependent on the degree of structural rearrangements for this particular aptamer–target interaction. Moreover, MST experiments with an ATP-binding aptamer carried out by Entzian and Schubert also led to a comparable amplitude (amplitude = 5 AU), and binding could be verified [24].

MST experiments at room temperature were not successful (data not shown), and no binding could be observed. However, a binding curve could be obtained when the MST chamber was heated to 35 °C, which is considerably closer to the melting temperature of the aptamer. Heating the system therefore shifted the equilibrium of the aptamer structure to an unfolded or at least less structured state, where the aptamer was not correctly folded. Upon target molecule addition, the aptamer folded into its native form, which resulted in a significant structural change that could be monitored by MST. 

Cholecalciferol was used as a negative control in another MST experiment to investigate the specificity of the aptamer. When using the 25(OH)D precursor vitamin D_3_, no binding curve could be observed. This showed the ability of the aptamer to distinguish between the 25-OH groups of the target molecule 25(OH)D. As far as we are aware, this high specificity has not yet been reported in the literature. This negative control was important to verify, since the product vitamin D_3_ is also present in clinical samples. These usually consist of a mixture of different pre-hormones of 25(OH)D and vitamin D_3_ [3]. The next step involved the design of complementary oligonucleotides for the desired TID assay format. The oligonucleotides needed to be designed to compete with the target molecule 25(OH)D. Therefore, when 25(OH)D was bound, the oligonucleotide would be released from the aptamer, which would result in a significant change in structure and thus an increase in the resulting amplitude in the MST experiments.

### 4.3. Design and Evaluation of Complementary Oligonucleotides

It was crucial to design the complementary oligonucleotides (which form duplexes with the aptamer) in a way that maximized the oligonucleotide dissociation, since a high signal-to-noise ratio is preferred. Three complementary oligonucleotides with a length of 10 nt were designed to cover the hairpins and stems in the predicted secondary structure based on the experimental results of the recorded CD spectra. The CD spectroscopy indicated that the target might bind to a preformed structural motif of the aptamer. In other published competitive assays, we have already been able to successfully use the 10-nt length [15,20]. Moreover, it could be shown that longer oligonucleotides lead to decreased complementary strand displacement and consequently decreased sensing performance [25]. The oligonucleotides were labeled 5′-Cy5, and the 25(OH)D-induced dissociation was analyzed using MST. It could be observed that O1 exhibited the smallest amplitude, followed by oligonucleotides 2 and 3. As reported by Jerabek-Willemsen et al., it is possible to conclude binding sites by observing the resulting MST amplitudes and comparing them in competitive experiments [26]. In this experiment, O2 and O3 showed the largest amplitude (O2 = 11, O3 = 35.55, see Table A1), which hinted at the most probable binding site of 25(OH)D (located in the S1 area between L1 and L2). Since O3 was the most promising candidate for further experiments in terms of amplitude, the MST experiment involving O3 was optimized, since *K_d_* confidence was rather high (Table A1). The variation in aptamer and O3 concentration (from 50 nM to 10 nM) led to an improved MST curve with an adequate *K_d_* confidence. On the basis of these findings, a competitive assay for the detection of the small molecule 25(OH)D was established using O3.

### 4.4. Biosensing Experiments Using the Established Aptasensor

The aim of this work was to develop an aptamer-based detection system using a target-induced dissociation mode that can be transferred to biosensors. In the previous steps, the general properties of the used aptamer were investigated, and a putative binding site of 25(OH)D was identified. As a final step, the VDBA14 aptamer was immobilized on glass slides, and experiments with a serial dilution of 25(OH)D and O3 were carried out. Aptamer saturation of the 3D aldehyde surface was observed at 12.5 μM of aptamer. The *K_d_* value of the immobilized aptamer can vary drastically from free aptamers due to steric hinderance and structural dislocation of the aptamer-binding sites [27]. High aptamer immobilization densities could lead to the inability to form secondary structures that are crucial for binding of the target [28]. Therefore, the binding ability of the immobilized aptamer was investigated in the next step using a dilution series of 25(OH)D. We tested two aptamer concentrations (6.25 and 12.5 µM) to investigate the effect of the immobilization density on the aptamer-binding capabilities. As expected, the oligonucleotide hybridization decreased with a rising 25(OH)D concentration. The maximal reduction of oligonucleotide hybridization could be observed at 2 μM of 25(OH)D at all tested aptamer concentrations (reduction of oligonucleotide hybridization = 78%). Since the 25(OH)D concentration range in this experiment was relatively broad (0–12 μM), the experiment was repeated in the lower nanomolar concentration range (0–50 nM 25(OH)D). As in the previous experiment, the displacement of the complementary O3 could be observed, starting at a 25(OH)D concentration of 12 nM. No displacement could be observed in the concentration range between 1 and 6 nM 25(OH)D. In general, 25(OH)D levels ≤50 nM are defined as a vitamin D deficiency and can lead to serious health conditions [4]. On the basis of the determined LOD (5.4 nM) and LOQ (18 nM), the established aptasensor was able to detect 25(OH)D concentrations present at critical vitamin D deficiency conditions in binding buffer. Microarrays should be viewed as a model platform, since they enable the highly parallel optimization of different parameters, such as aptamer concentration and oligonucleotide concentration. Additionally, microarrays enable the potential for multiplexing and the simultaneous analysis of different analytes. On the basis of these first microarray experiments, the current detection system can be applied to more cost-efficient transduction methods, e.g., optical methods and a corresponding sensor system can be established.

## Appendix A

**Table A1:** Estimated apparent dissociation constants, amplitudes, and *K_d_* confidence of the complementary oligonucleotides before optimization was carried out. Apparent *K_d_* values are stated with “≥” since an exact determination of *K_d_* is only possible when the binding curve reaches a plateau at high concentrations. In case the plateau is not reached, the binding curves can only be used to determine the lowest possible *K_d_*, while the real value could be higher.

Oligonucleotide	Amplitude (AU)	Apparent *K_d_* (nM)	*K_d_* Confidence (nM)
1	3.45	≥50	±6.57
2	11.94	≥100	±3.46
3	35.55	≥660.91	±801.61
